# Geographic Variation of Strontium and Hydrogen Isotopes in Avian Tissue: Implications for Tracking Migration and Dispersal

**DOI:** 10.1371/journal.pone.0004735

**Published:** 2009-03-06

**Authors:** Megan J. Sellick, T. Kurt Kyser, Michael B. Wunder, Don Chipley, D. Ryan Norris

**Affiliations:** 1 Department of Integrative Biology, University of Guelph, Guelph, Ontario, Canada; 2 Department of Geological Sciences and Geological Engineering, Queen's University, Kingston, Ontario, Canada; 3 Department of Biology, University of Colorado Denver, Denver, Colorado, United States of America; University of Bristol, United Kingdom

## Abstract

**Background:**

Isotopes can provide unique solutions to fundamental problems related to the ecology and evolution of migration and dispersal because prior movements of individuals can theoretically be tracked from tissues collected from a single capture. However, there is still remarkably little information available about how and why isotopes vary in wild animal tissues, especially over large spatial scales.

**Methodology/Principal Findings:**

Here, we describe variation in both stable-hydrogen (δD_F_) and strontium (^87^Sr/^86^Sr_F_) isotopic compositions in the feathers of a migratory songbird, the Tree Swallow (*Tachycineta bicolor*), across 18 sampling sites in North America and then examine potential mechanisms driving this variation. We found that δD_F_ was correlated with latitude of the sampling site, whereas ^87^Sr/^86^Sr_F_ was correlated with longitude. δD_F_ was related to δD of meteoric waters where molting occurred and ^87^Sr/^86^Sr_F_ was influenced primarily by the geology in the area where feathers were grown. Using simulation models, we then assessed the utility of combining both markers to estimate the origin of individuals. Using 13 geographic regions, we found that the number of individuals correctly assigned to their site of origin increased from less than 40% using either δD or ^87^Sr/^86^Sr alone to 74% using both isotopes.

**Conclusions/Significance:**

Our results suggest that these isotopes have the potential to provide predictable and complementary markers for estimating long-distance animal movements. Combining isotopes influenced by different global-scale processes may allow researchers to link the population dynamics of animals across large geographic ranges.

## Introduction

Understanding the ecology, evolution, and life-history strategies of animals requires detailed knowledge of individual movements throughout the year [1], [2]. Uncovering patterns of migration and dispersal has been challenging for many species because of the difficulty associated with tracking the movements of individuals over large geographic distances [1]–[3], many of which cover thousands of kilometres. Marked individuals are rarely recaptured [4], [5] and satellite tags are still too large for many smaller species (Microwave Telemetry, Inc., Columbia, MD, USA).

Isotopes of specific elements provide a potential solution to the challenges associated with estimating animal movements because individuals only need be captured once to estimate the origin of selected tissues grown during a previous season [3], [6]–[9]. Animals incorporate isotopic signatures into their tissues through local diet sources and, depending on the turnover rates within tissues (days to weeks in blood and liver: [10], [11]; up to a year in bone tissue: [10]), samples from individuals in one period of their life cycle can be used to infer their origin from the period in which the tissue was formed. Successful application of this technique relies partly on predictable geographic variation of the isotopic composition of a give element [7], [12]. For example, stable-hydrogen isotopic compositions (δD) in animal tissues are closely related to δD values in precipitation (δD_P_; [6], [7], [13], [14] and δD_P_, in turn, varies with latitude according to elevation and meteorological patterns [12], [14], [15]. Several studies have exploited the geographic distribution of δD_P_ values to estimate the locations of migratory birds during different periods of the annual cycle [6], [7], [9], [13], [16]–[18].

To date, the assignment of individuals to specific geographic locations using δD has been limited to coarse regional scales. Reasons for this are not entirely clear but are likely influenced, in large part, by variation in temperature, elevation, meteorological storm patterns, and individual physiology, which, in turn, lead to local spatial and temporal variation of δD values [19]–[22]. One oft-mentioned solution for increasing the resolution of assignments is to use multiple isotopes [1], [3], [8], [23]. Thus far, efforts to incorporate multiple isotopic markers have achieved only limited success largely because new markers have not always provided complimentary information that increases the resolution for geographic assignment [19], [24], [25]; [but see 23], [26].

The isotopic ratio of the heavy element strontium (^87^Sr/^86^Sr) is one marker hypothesized to serve as a useful signal of geographic origin [6], [8], [27], [28]. Strontium (Sr, atomic number 38) is a non-nutrient, alkaline earth metal whose isotopic ratio of ^87^Sr/^86^Sr changes with time because of the decay of ^87^Rb, an unstable isotope of rubidium (Rb) to ^87^Sr [29]. As a result, the radiogenic isotope ^87^Sr increases relative to the stable ^86^Sr and ^88^Sr isotopes such that the highest ^87^Sr/^86^Sr ratios are generally found in older bedrock [29]. ^87^Sr/^86^Sr has been used to estimate short-distance movements of salmonids (Salmonidae; [30]), to characterize African Elephant (*Loxodonta Africana*) populations [31], and to estimate the migratory movements of extinct megafauna [32], [33]. In birds, Chamberlain *et al.* [6] combined ^87^Sr/^86^Sr ratios with δD and stable-carbon isotopes (δ^13^C) to infer the breeding area of Black-throated blue warblers (*Dendroica caerulescens*) sampled on their Caribbean wintering grounds. However, tissue sampling was limited to a small number of sites in the eastern U.S. and ^87^Sr/^86^Sr analysis was conducted on bone, which may have integrated ^87^Sr/^86^Sr ratios from diet consumed over multiple periods of the year. Thus, we still do not have a clear understanding of how ^87^Sr/^86^Sr varies across large geographic scales in a single season or the mechanisms driving this variation.

**Figure 1:**
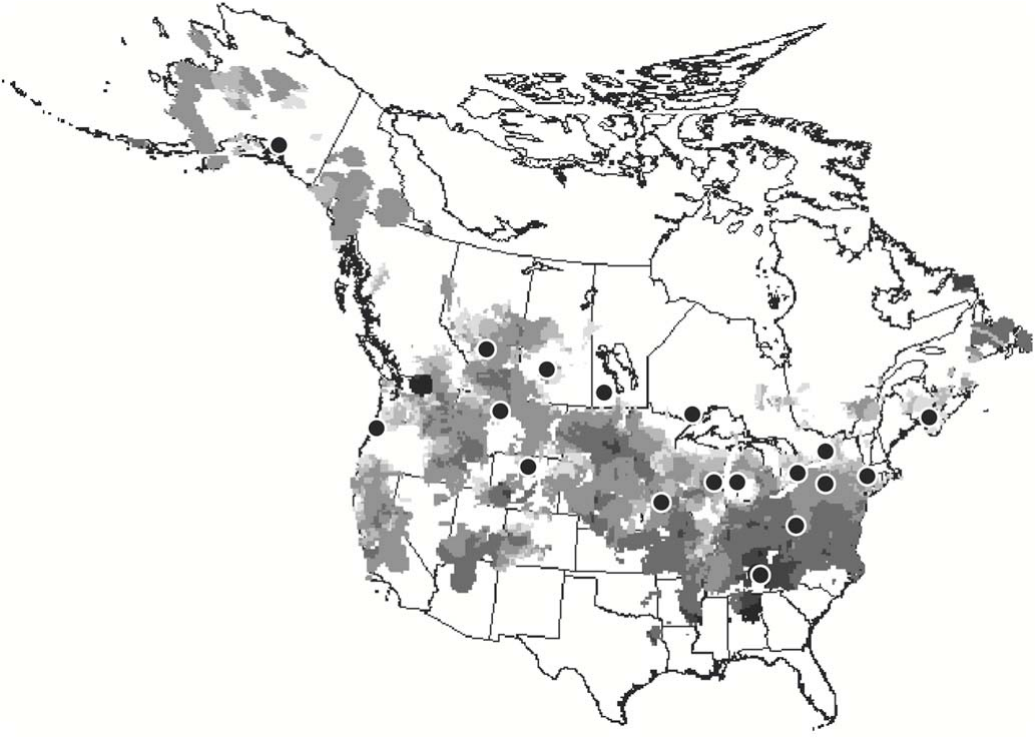
Map of 18 Tree Swallow sampling sites. Sites are black dots, which are overlaid on the relative breeding abundance (based on data from the Breeding Bird Survey; [37]). The intensity shading represents breeding density where lightest grey is lowest density (<1 individual) and black is the highest density (>100 individuals; following [37]).

Here, we examined the geographic variation and potential causes of such variation in both ^87^Sr/^86^Sr and δD in feathers of a migratory songbird, the Tree Swallow (*Tachycineta bicolor*), grown at 18 breeding sites across North America (figure 1). Because feathers are metabolically inert after growth, their isotopic signature represents the location of feather growth the previous breeding season [34]. First, we examined the relationship between δD in feathers (δD_F_) and δD in precipitation, as estimated from spatially interpolated values (δD_GS_; [12], [14]). Based on previous studies [6], [7], [23], [35], we predicted (a) a positive relationship between δD_F_ and δD_GS_, (b) that the intercept of this relationship would be in the range of −27‰ and −19‰ [14], [23], [35] and (c) that the slope of the relationship would not differ significantly from 1. Because deuterium tends to decrease with latitude, we also predicted that δD_F_ would be negatively correlated with the latitude at which feathers were grown. Because the age of underlying bedrock is one factor hypothesized to influence ^87^Sr/^86^Sr ratios [36]_,_ we predicted a positive relationship between ^87^Sr/^86^Sr in feathers (^87^Sr/^86^Sr_F_) and bedrock age. To explore whether there was a relationship between ^87^Sr/^86^Sr_F_ ratios and geographic location, we also examined correlations between ^87^Sr/^86^Sr_F_ and both latitude and longitude of the breeding site. Lastly, we developed a simulation model to test whether the combination of ^87^Sr/^86^Sr_F_ and δD_F_ would increase the probability of correctly assigning individuals to their site of origin over using either isotope alone.

## Methods

All animals were handled in strict accordance with good animal practice as defined by the relevant national and local animal welfare bodies, and all animal work was approved by the appropriate committees.

### Study species and sampling sites

Tree Swallows are small (21 g) insectivorous migratory passerines that breed throughout North America ([37]; figure 1) and nest almost exclusively in human-made wooden boxes [38]. From April–July 2007, we sampled feathers from adult birds at 18 breeding sites (figure 1, appendix S1) from the *Golondrinas de las Americas* network (David Winkler, Cornell University, Ithaca, NY; http://golondrinas.cornell.edu/). Sample sites were selected to include a range of distinct bedrock geologies (*e.g.* Proterozoic complex: northwest Ontario, Mesozoic sediments: Manitoba, and Cenozoic volcanics: Oregon) and cover a range of known δD values in precipitation from Alaska to southeastern US [12], [14], while ensuring samples could be collected in a single season to eliminate year effects. We collected samples from 14 sites and received samples from colleagues at the other four (Wolfville, NS; Amherst, MA; Portland, OR; McCarthy, AK).

### Tissue sampling

Although feather of nestlings would be logistically easier to sample, we chose to sample adult feathers because substantial differences in δD between adult and nestling from the same site have been reported in other songbirds [22], as well as Tree swallows (G. Betini, K.A. Hobson, L.I. Wassenaar, DRN, unpublished data). To trap adults, we used several live-capture methods (*e.g.* moe-traps, flap-traps, wig-wag traps) while they were incubating eggs or feeding young. Upon capture, we determined sex based on the presence of a brood patch (only females incubate) and female age based on plumage colour and iridescence [39]. We then clipped the first primary feather (P1) approximately 0.5 cm from its base. The site at McCarthy, AK was the exception to this protocol. The only available samples were P1s from two recently deceased fledglings.

To maximize the probability that ^87^Sr/^86^Sr and δD values in feathers were representative of the site in which they were sampled, we analyzed the P1 from marked adults known to have bred at the same site the previous year (*n* = 6 sites). Tree Swallows undergo a complete pre-basic molt (all feathers) on the breeding ground prior to fall migration. Primary and secondary flight feathers are generally molted from the inside outward, beginning as early as July [39], [40]. If it was not possible to sample marked individuals, we sampled after-second-year (ASY) females (*n* = 9 sites) because previous studies have shown that they have higher site fidelity than second-year (SY) females [38]. With the exception of two sites (NW ON [Thunder Bay] and SW ON [Guelph]), we did not sample males that were unbanded because it was difficult to determine age beyond simply classifying them as after-hatch-year (AHY).

### Isotope analysis

We analyzed samples at the Queen's Facility for Isotope Research, Queen's University, Kingston, Ontario. Feathers were first soaked in a 2∶1 solution of chloroform: methanol for 24 hrs to remove surface oils, then allowed to air dry for 36 hrs.

#### Stable-hydrogen isotope (δD) analysis

Stable-hydrogen isotope ratios (^2^H/^1^H = R) are expressed in δ units where δ = [(R_sample_/R_standard_) − 1]×1000. From each feather, we sub-sampled 0.1–0.4 mg of tissue for δD analysis. Sub-samples were left open in the lab for 72 hrs to allow the feather's exchangeable hydrogen to equilibrate to the lab environment before analysis. 0.1–0.15 mg of each feather sample was loaded into 3×4.2 mm silver capsules and left in a 100°C oven to outgas overnight to minimize the effect of exchangeable hydrogen. In-house standards were loaded into silver capsules in the same way and left in a 100°C oven for 1 hr. Silver capsules were then sealed, loaded into a ThermoFinnigan TCEA auto-sampler, and introduced on-line to a ThermoFinnigan Delta Plus XP Mass Spectrometer through a Conflo III Interface. One standard was run for every 5–8 unknowns and a duplicate unknown was run every 8–10 samples to verify accuracy of results. During analysis, three in-house standards were run (mean±s.d.): Georgia Clay (−58‰±2, *n* = 19), UofM Brucite (−93‰±5, *n* = 21), and a Blue Jay (*Cyanocitta cristata*) feather (−47‰±5, *n* = 6). All standards matched previous values in the lab. The mean±SD difference between duplicate (same feather) analyses of unknown samples was 3.2‰±2.8 (*n* = 17).

#### Strontium isotope (^87^Sr/^86^Sr) analysis

We digested the remaining feather samples (4.8–10.3 mg) in 2–3 mL concentrated nitric acid in Savillex Teflon sample vials on a 70°C hotplate for approximately 3 hrs. Samples were then cooled for 1 hr before the addition of 30% hydrogen peroxide (H_2_O_2_; 0.5 mL) to digest remaining organic material. Two hours after the addition of H_2_O_2_, capped vials were returned to the 70°C hotplate for 3–4 hrs. Caps were then removed to allow the solution to dry down on the hotplate overnight. Dried samples were acidified with 3 g of 3 M nitric acid and allowed to sit covered until fully dissolved (up to 6 hrs). One gram of this sample was then loaded into inert column supports filled with Eichrom's Sr Spec Resin (1.0 M 4,4′(5′)-di-t-butylcyclohexano 18-crown-6 (crown ether) 1-Octanol). This resin retains strontium within the crown ether at high nitric acid concentrations, while allowing other elements and compounds to pass through. The crown ether releases strontium at a concentration of nitric acid below 0.05 M (Eichrom Technologies, Inc.). Before adding digested and acidified samples to the columns, the resin was cleaned and equilibrated with 3 M nitric acid and de-ionized water. Acidified feather samples were loaded into the columns (500 µL, 4 times) before flushing the columns five times with 500 µL of 3 M nitric acid to elude most elements with the exception of strontium and lead. Strontium was released from the column, using 500 µL 0.05 M nitric acid, and collected in a 2 mL Teflon vial. These samples were dried on a 70°C hotplate overnight and acidified with 2 g of 2% nitric acid, transferred to clean Teflon sample vials, and loaded into the auto-sampler of the ThermoFinnigan Neptune high-resolution multicollector ICP-MS for measurement of the ^87^Sr/^86^Sr ratio.

For the ^87^Sr/^86^Sr ratio, the MC-ICP-MS takes 63 consecutive measurements per sample. For each sample, seven atomic masses were measured: ^84^Sr, ^86^Sr, ^87^Sr, ^88^Sr, and ^90^Sr, as well as ^83^Kr, ^85^Rb. The Sr content was adjusted for both the standards and samples to 10 ppb, 5 ppb or 2 ppb depending on the amount of strontium in the sample and each sample was bracketed with standards. All ratios were normalized to ^86^Sr/^88^Sr to account for mass fractionation and all results were corrected for blanks. The non-strontium masses are measured to account for potential sources of contamination in ^87^Sr/^86^Sr ratios, including Kr from the plasma gas, ^87^Rb corrected using ^85^Rb and Ca diamers using the ^45^Ca diamer to reflect these. ^87^Sr/^86^Sr ratios are expressed as the mean of 63 measurements. The mean SE of unknown samples was 0.00004. The National Institute of Standards and Technology certified NBS 987 produced a mean (±s.e.) ^87^Sr/^86^Sr ratio of 0.71025 (±0.00002, *n* = 31) for 10 ppb Sr, 0.71025 (±0.00004, *n* = 7) for 5 ppb, and 0.7102 (±0.0002, *n* = 13) for 2 ppb. Strontium isotope ratios are expressed as the ratio of ^87^Sr to ^86^Sr and the ^87^Sr/^86^Sr ratio was corrected using a ratio of ^86^Sr/^88^Sr (0.1194). The mean (±s.d.) of the in-house organic standard (Wild turkey feather, *Meleagris gallopavo*) was 0.7086±0.0006 (*n* = 4).

### GIS kriging

To visually illustrate the spatial variation of each isotope, we used ArcView 9.2 (ESRI, Redlands, California, USA) to create a series of contour surfaces based on the mean δD_F_ value and ^87^Sr/^86^Sr_F_ ratio at each of the 18 sites. We used ordinary kriging to assign weights to data points within a neighbourhood, which was defined as the region of search around the location to be interpolated [41], [42]. The purpose of these maps was to provide a visual description of spatial variation and was not used for assigning birds to specific areas in North America.

### Statistical analysis

We used Spearman's rho correlation (*r_s_*) to examine the relationship between the geographic location of each sampling site (latitude and longitude) and the mean isotope values of feathers (δD_F_ and ^87^Sr/^86^Sr_F_) sampled at each site. We used generalized linear mixed-effects models (GLMM) with restricted maximum likelihood protocol (REML) in R (version 2.6.1, R Core Development Team) to investigate the potential mechanisms driving the patterns of variation in δD_F_ values (δD in precipitation) and ^87^Sr/^86^Sr_F_ (age of bedrock) in avian feathers. Mixed-effect models included site as a random-effect to account for individual differences at each sampling site.

For δD values in precipitation, we used site-specific, spatially interpolated growing season precipitation (δD_GS_) values from the OIPC (Online Isotopes in Precipitation Calculator; www.waterisotopes.org). These values were used instead of analysing δD values in actual water samples because the δD value of standing water (*i.e.* ponds and lakes) are predicted to change substantially as the growing season progresses [43]. Therefore, water collected during the short window of time when we visited each breeding site (3–6 days) would likely not have reflected the δD values being assimilated into plants, insects, and ultimately birds throughout the breeding season (2–3 months). Bedrock ages were derived by determining the geologic classification of the bedrock (*e.g.* Phanerozoic complex, Mesozoic complex) using the Global GIS Database v. 6.2, U.S. Geological Survey, Flagstaff, AZ, USA (www.agiweb.org/pubs/). We then determined the estimated age of the components that make up the rocks in the area that contribute to the strontium reservoir of the region (see appendix S2). The age of unsorted glacial material (till) was estimated from the age of the bedrock in the region and known glacial direction of transport. Values of estimated age of bedrock were log-transformed to meet normality requirements. For both mechanistic model types (precipitation and age of bedrock), we evaluated the strength of mixed-effects versus fixed-effects models using Akaike's Information Criterion for small sample sizes (AIC_c_) and evidence ratios (ER = e^0.5*ΔAICc^; [44].

To compare with previous studies, ordinary least squares (OLS) regression was used to estimate the discrimination factor (intercept) and slope of the δD_F_∼δD_GS_ relationship. Because we used the mean δD_F_ value from each site for this analysis, we emphasize that this was only done to compare our intercept with prior work. Normality of within-group errors was tested by examining normal probability plots of the random effects and residuals for each model. Heteroscedasticity of the data was assessed by examining the plots of standardized residuals versus fitted values.

### Assignment simulations

We performed a series of simulations to test whether the combination of the two isotopes would increase the probability with which individuals were correctly assigned to sites of origin when compared to using each isotope alone. The simulation model was used because it is robust in cases with small sample sizes and is more appropriate for comparing the number of correct assignments in dual- versus single-isotope markers.

First, separate univariate normal probability distribution functions were fit for δD_F_ and ^87^Sr/^86^Sr_F_ values to feather data from each site and then 5000 isotope values were simulated for each site from the fitted probability density functions. Each simulated isotope value was then assigned back to one of the 18 sampling sites based on maximum likelihood. Because we knew which probability distribution function generated each synthesized datum, this procedure allowed us to calculate the percentage of correct assignments for each site independently and for all sites combined based on using either δD_F_ or ^87^Sr/^86^Sr_F_. We then repeated this procedure but combined ^87^Sr/^86^Sr_F_ and δD_F_ values by fitting bivariate normal distribution functions to the data for each site.

To explore whether each of the isotopes alone or together would be useful for assigning individuals to larger geographic areas, we reduced the number of potential breeding sites from 18 to 13 and repeated the above procedure. Sites were grouped by geographic proximity. In constructing all of the above models, we assumed that the actual data values of δD_F_ and ^87^Sr/^86^Sr_F_ were an accurate representation of a random univariate or bivariate normal distribution and that the analytical/measurement error was negligible (*i.e.* that each isotope value is known exactly; [45]). Although these models are more robust against small sample sizes, this approach is limited in its power to estimate the origin of individuals when samples sizes are particularly low (*i.e.* McCarthy, AK site: N = 2). However, our purpose in developing the models was simply to test whether 2 isotopes would be more effective than a single isotope. All simulations were preformed in Matlab (The MathWorks Inc., Natick, MA, USA).

## Results

### Geographic distribution of δD_F_ and ^87^Sr/^86^Sr_F_

**Figure 2:**
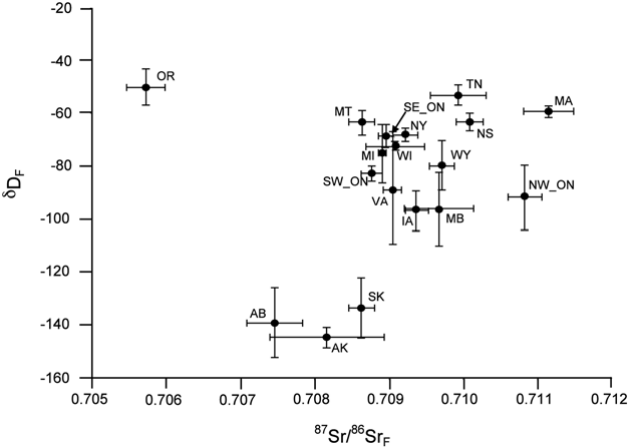
Relationship between δD and ^87^Sr/^86^Sr values in Tree swallows sampled across North America. Values are mean±s.e. from primary flight feathers. Abbreviations are: AB = Red Deer, Alberta; AK = McCarthy, Alaska; IA = Ames, Iowa; MA = Amherst, Massachusetts; MB = Brandon, Manitoba; MI = Allendale, Michigan; MT = Monarch, Montana; NS = Wolfville, Nova Scotia; NW ON = Thunder Bay, Ontario; NY = Ithaca, New York; OR = Portland, Oregon; SE ON = Elgin, Ontario; SK = Saskatoon, Saskatchewan; SW ON = Guelph, Ontario; TN = Lenoir City, Tennessee; VA = Waynesboro, Virginia; WI = Saukville, Wisconsin; WY = Big Horn, Wyoming.

δD values (±s.e.) in Tree swallow feathers (δD_F_) ranged from −50‰±7 (Portland, OR) to −145‰±4 (McCarthy, AK) and ^87^Sr/^86^Sr_F_ ratios (±s.e.) ranged from 0.7111±0.0003 (Amherst, MA) to 0.7057±0.0003 (Portland, OR; figure 2, appendix S1).

**Figure 3:**
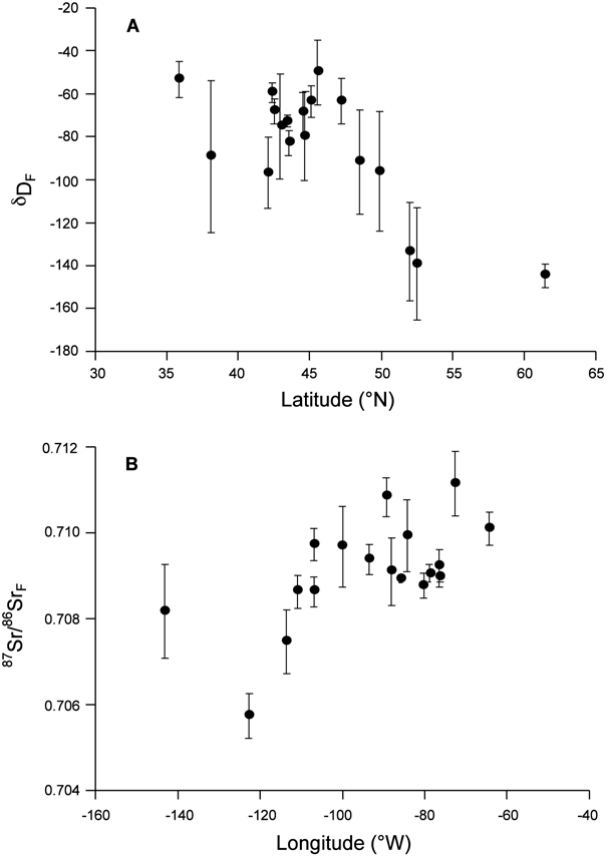
Relationship between geographic location and isotopes in flight feathers of Tree swallows. Isotope values are mean±s.d. from 18 sites. *(a)* latitude versus δD_F_ (*r_s_* = −0.47, *P* = 0.05), *(b)* longitude versus ^87^Sr/^86^Sr_F_ (*r_s_* = 0.62, *P* = 0.009).

**Figure 4:**
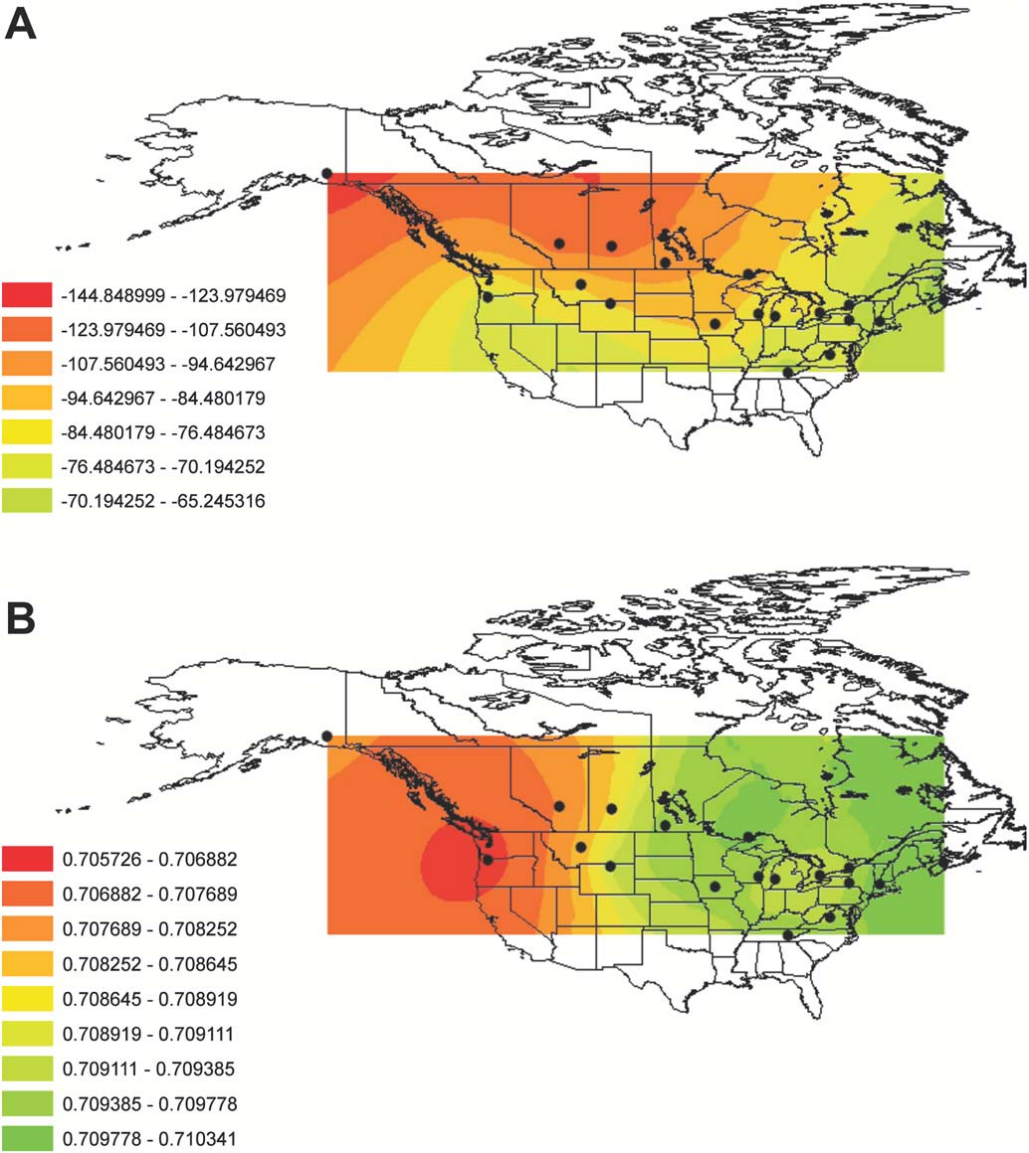
Geographic variation of *(a)* δD and *(b)* ^87^Sr/^86^Sr values in Tree Swallow feathers. Contour maps were produced by ordinary kriging and are based on mean values in primary flight feathers at 18 breeding sites (denoted by black circles).

As predicted, δD_F_ exhibited a negative correlation with breeding site latitude (*r_s_* = −0.47, *P* = 0.05; figure 3*a*), whereas there was a weaker correlation with longitude (*r_s_* = 0.41, *P* = 0.09, *n* = 18). Conversely, ^87^Sr/^86^Sr_F_ was positively correlated with longitude (*r_s_* = 0.62, *P* = 0.009; figure 3*b*) and there was a weaker negative correlation with latitude (*r_s_* = −0.45, *P* = 0.06, *n* = 18). Consistent with these isotope providing complimentary information, there was no correlation between the mean values of δD_F_ and ^87^Sr/^86^Sr_F_ in Tree swallows across sampling sites (*r_s_* = 0.13, *P* = 0.26, *n* = 79, figure 2). Interpolated maps of both isotopes illustrate the geographic variation of both δD_F_ (figure 4*a*) and ^87^Sr/^86^Sr_F_ (figure 4*b*) across North America.

### Mechanisms of geographic variation in δD_F_ and ^87^Sr/^86^Sr_F_

**Figure 5:**
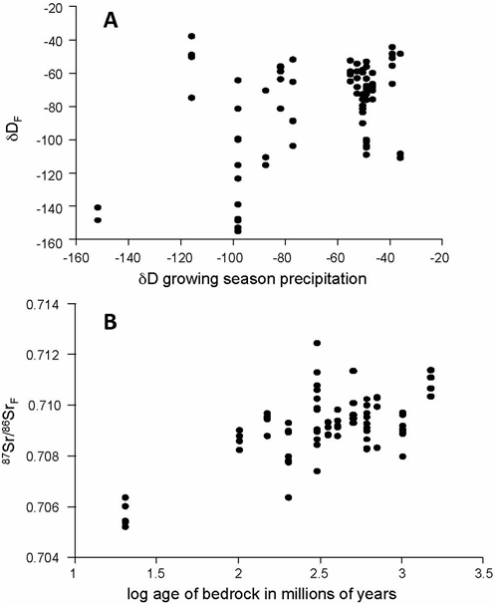
Predictors of δD and ^87^Sr/^86^Sr values in Tree swallow primary flight feathers. Each point represents an individual (*n* = 79). *(a)* δD growing season precipitation (δD_GS_) versus δD_F_ *(b)* age of under lying bedrock versus ^87^Sr/^86^Sr_F_. δD_GS_ data are from waterisotopes.org [14]. Bedrock ages represent the estimated age of the components that make up the rocks in the area that contribute to the strontium reservoir of the area (see appendix S2). Results from the GLMM are presented in table 1.

As predicted, we found that Tree swallows with more positive δD_F_ values tended to be from breeding sites with more positive δD_GS_ (table 1, figure 5a), and that individuals with higher ^87^Sr/^86^Sr_F_ ratios tended to be from breeding sites with older underlying bedrock (table 1, figure 5*b*). In both cases, the mixed-effect model had greater strength of evidence relative to the fixed-effect model (lower ER values; table 1).

**Table 1:** Results from the generalized linear mixed effects models (GLMM) used to examine the mechanisms influencing δD_F_ and ^87^Sr/^86^Sr_F_.

	slope estimate	s.e.	*r^2^*	AIC_c_	ΔAIC_c_	e.r.
δD_F_∼δD_GS_, mixed	0.51	0.19	0.67	714	0	1
δD_F_∼δD_GS_, fixed	0.44	0.11		751	37	1.1×10^8^
^87^Sr/^86^Sr∼bedrock, mixed	0.00032	0.00008	0.84	−881	0	1
^87^Sr/^86^Sr∼bedrock, fixed	0.00032	0.00004		−831	50	2.4×10^12^

The relative performance of the mixed-effects versus fixed-effects models was assessed using the difference in the Akaike's Information Criterion for small sample sizes (ΔAIC_c_) and evidence ratios (ER = e^0.5*ΔAICc^). Mixed effect models included sample location as a random effect in addition to the fixed effect (δD growing season precipitation [δD_GS_] for δD_F_ and log transformed bedrock age [bedrock] for ^87^Sr/^86^Sr_F_).

The intercept of the δD_F_∼δD_GS_ relationship using OLS regression was −47‰±14, which was substantially lower than values from previous studies (*e.g.* −6 to −31‰; [7], [14], [16], [20], [23], [35] [but see 19]). Also unlike previous studies (*e.g.* [7], [14], [16], [20], [23], [46]), the slope of the δD_F_∼δD_GS_ relationship was less than 1 (*β* = 0.52±0.19). When we constrained the slope to 1 and re-ran the model, the sum of squares and *R^2^* were both 0, suggesting that the slope was significantly different than 1.

### Assignment tests

**Figure 6:**
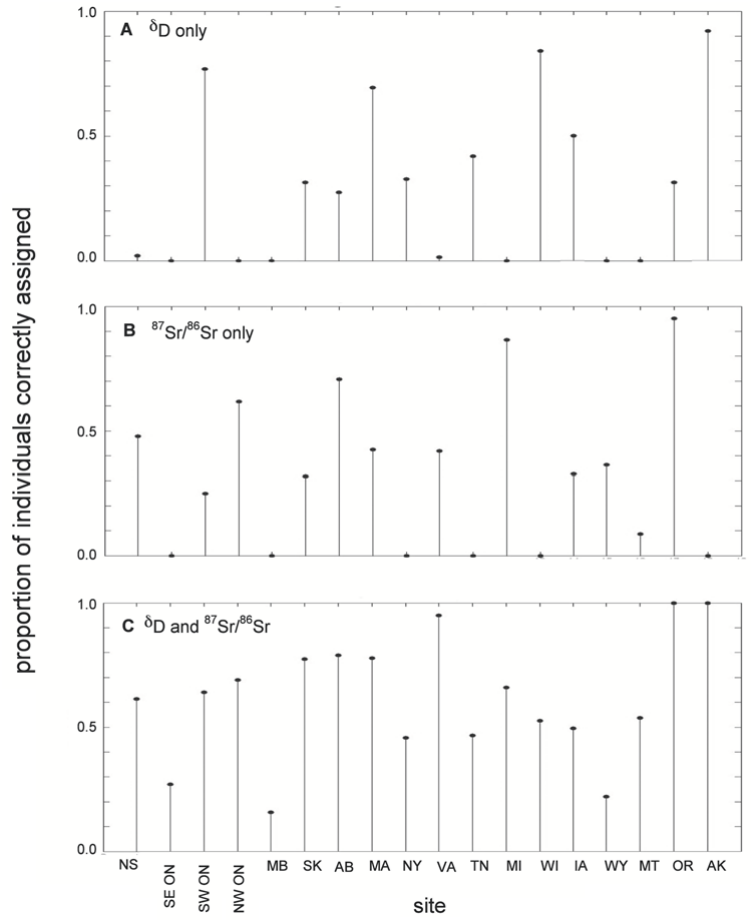
The proportion of Tree swallow correctly assigned to their original site of origin. *(a)* δD, *(b)* ^87^Sr/^86^Sr, and *(c)* δD and ^87^Sr/^86^Sr. Proportion of correct assignments are based on 5000 simulations at each of the 18 breeding sites.

Using all 18 locations as potential sites of origin, we found that only 30% of individuals were correctly assigned to their breeding site of origin using δD alone (figure 6*a*) and only 32% when using ^87^Sr/^86^Sr alone (figure 6*b*). When both isotopes were used alone, only 17% (3/18) of the sites had a correct assignment rate greater than 70% and only one had a rate greater than 90%. However, when δD and ^87^Sr/^86^Sr were combined, 61% of individuals were correctly assigned to their site of origin (figure 6*c*) and 33% (6/18) of the sites had a correct assignment rate greater than 70% (3 sites had greater than 90%).

**Figure 7:**
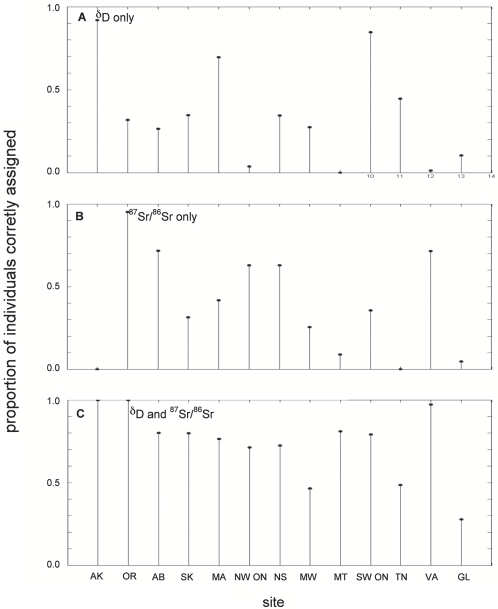
The proportion of Tree swallows correctly assigned to their original site of origin. Same as figure 6, except 18 sites were collapsed to 13 sites based on geographic proximity. The Great Lakes group (GL) includes Ithaca, NY, Elgin, ON, Allendale, MI, and Saukville, WI, the Mid-west group (MW) includes Ames, IA, Big Horn, WY, and Brandon, MB. All other sites are the same as in figure 6.

When we collapsed the number of potential sites of origin from 18 to 13, we found that 35% and 39% of individuals were correctly assigned using δD and ^87^Sr/^86^Sr, respectively (figure 7*a,b*). When both isotopes were used alone, only 23% (3/13) of the sites had correct assignment rates greater than 70% and one was greater than 90%. In contrast, 74% of individuals were correctly assigned to their site of origin when δD and ^87^Sr/^86^Sr were combined (figure 7*c*) and 77% (10/13) of the sites had greater than 70% of correct assignments (3 sites had greater than 90%).

## Discussion

Achieving greater resolution with multiple isotopes is critical for being able to estimate the origins and movements of animals that are nearly impossible to follow using traditional methods. Our results provide evidence that ^87^Sr/^86^Sr and δD can be used as complementary geographic markers to estimate the origins of animals in North America. We found that δD values in Tree swallow feathers (δD_F_) were correlated with latitude whereas ^87^Sr/^86^Sr ratios in feathers (^87^Sr/^86^Sr_F_) were related to the longitude of the sampling site. Simulation results demonstrated that combining these markers more than doubled the percentage of individuals successfully assigned to their sites of origin when compared to using either isotope marker alone.

Our study also provides the first test of the hypothesis that ^87^Sr/^86^Sr ratios in birds are related to the age of underlying bedrock across large geologic scales. Even when one outlying site was removed (Portland, OR) the relationship remained strong (GLMM: *R^2^* = 0.71, *β* = 0.001±0.0007, *α* = 0.707±0.0018). Although bedrock age was a significant predictor of ^87^Sr/^86^Sr_F_, other factors likely contribute to variation in ^87^Sr/^86^Sr ratios in animal tissues. For instance, the precise geochemistry of local bedrock is likely to influence Rb/Sr ratios in the food web. For example, two of the sites (Wolfville, NS and McCarthy, AK) had the same estimated bedrock age (300 my) but significantly different mean ^87^Sr/^86^Sr_F_ ratios (1-tailed t-test; *t*_5_ = −3.82, *P* = 0.006). The integrated Rb/Sr ratios of the sedimentary rocks around Wolfville are higher than those of those of igneous rocks in Alaska, likely resulting in higher bedrock ^87^Sr/^86^Sr ratios. Predicting precise ^87^Sr/^86^Sr ratios in animal tissues will require integration of the geological histories of local areas.

Although analytical variability in δD is known to be a significant source of error when estimating animal movements [45], there have been no studies that have reported repeatability of ^87^Sr/^86^Sr in animal tissues. We found that the coefficient of variation in δD from repeated measurements of in-house feather standards (CV = 0.12) was much higher than ^87^Sr/^86^Sr (CV = 0.009). One reason for this is that stable peak signal intensities are measured in Sr ratios rather than integrating the total intensity, as in δD analysis. A second reason is that, aside from diet, δD values in tissues are influenced by a range of metabolic processes [47] because of the large difference in the masses of hydrogen isotopes and the different bonding states that could contribute to substantial variation. Strontium, in contrast, is a non-nutrient mineral and the bonding environment is relatively constant. Despite this, we still recommend that future studies asses the influence of Sr variability when assigning animals to geographic locations [45], [48].

Although our results suggest that the δD_GS_ is a useful predictor of δD_F_, the slope of the δD_F_∼δD_GS_ relationship (*β* = 0.52±0.19) was smaller compared to previous studies (*e.g. β* = 0.68 to 1.0: [7], [14], [16], [20], [46]. A slope of less than one implies that large differences in δD_GS_ values across the landscape will result in only small differences in δD_F_ values. Potential reasons for a slope less than 1 include differences between sites in available diet, rates of evaporation, seasonal temperature trends, or the possibility that δD_GS_ estimates could be incorrect for the sampled areas. Future work is needed to examine the mechanisms causing variation in the slope of this relationship. Otherwise, it is not possible to use corrected δD_GS_ values for assigning animals to geographic locations without significant amounts of error.

Several factors may account for the unexplained variation in the δD_F_∼δD_GS_ relationship. First, interpolated δD_GS_ values were derived from IAEA stations in North America with limited geographic coverage [14], [45]. Second, limited access to water can cause individuals to become deuterium enriched due to high rates of water loss [49] and Tree swallows varied considerably in their proximity to bodies of water [38]. We regressed the residuals from the δD_F_∼δD_GS_ regression against distance to water and found that individuals further away from water tended to be more enriched in deuterium than individuals closer to water (*β* = 1.8, *P* = 0.03), although the amount of variation explained was low (*R^2^* = 0.06).

Differences in proximity to water between species may also explain why the discrimination value (−47‰±14) between water and tissue (intercept of the δD_F_∼δD_GS_ relationship) was lower and than in previous studies ([7]: −31‰; [46]: −26‰; [16]: −34‰; [35]: −25‰; [14]: −19‰; [20]: −6‰). The difference between δD_F_ and δD_GS_ could be partly driven by the fact that there is likely large variation in water loss among Tree swallows. In support of this hypothesis, we found that the discrimination value for Tree swallows sampled at sites greater than 3 km from water was more positive (−28‰, *R^2^* = 0.51, *β* = 0.60, *n* = 4), and similar to that of previous studies, compared to the discrimination value for individuals sampled 0–2 km from water (−52‰, *R^2^* = 0.35, *β* = 0.51, *n* = 14).

Although we attempted to sample tissues of known-origin, it is possible that not all birds grew their feathers at the site they were sampled. Forty-two percent of the Tree swallows we sampled were known to have bred at the same site the previous year. If a large percentage of the remaining unmarked birds immigrated from other populations, then sites with only unmarked individuals (*n* = 10) should have greater variation in ^87^Sr/^86^Sr_F_ and δD_F_ than sites with only marked birds (*n* = 7; one site where both marked and unmarked birds were present was excluded). However, we found no difference in the variation between these groups (1-tailed t-test; ^87^Sr/^86^Sr: *mean std. dev_unmarked_* = 0.0006, *mean std. dev_marked_* = 0.0005, *t_15_* = *−*0.743, *P* = 0.23, δD: *mean std. dev_unmarked_* = 17, *mean std. dev_marked_* = 14, *t_15_* = −0.44, *P* = 0.33).

In addition to only having to sample individuals once to infer their origin, stable-light isotopes are ideal for tracking long-distance movements of small animals because analysis only requires a small amount of tissue (as little as 0.15 mg) and the cost has decreased considerably over the last decade. However, these advantages may not be directly transferable to isotopic studies of heavy elements. Because strontium is less abundant in animal tissues, substantially more tissue is needed for analysis. Previous studies have used large quantities (up to 25 mg) of bone [5], although we were able to produce reliable ^87^Sr/^86^Sr ratios from approximately 5 mg of feather. A previous study was able to obtain ^87^Sr/^86^Sr ratios from feather samples using 0.2–1.5 mg [28], but they used Thermal Ionization Mass Spectrometry (TIMS) that is more laborious and lengthy than MC-ICP-MS. Even so, MC-ICP-MS costs are ten times that of light isotope analysis. This is due, in large part, to the maintenance of more specialized equipment and the materials and time required to extract pure strontium from digested feather samples. Although the cost per sample is decreasing, it is unlikely to match the current costs of light isotopes in the near future.

Our study suggests that combining heavy and light isotopes may provide the opportunity to link the dynamics of populations over large geographic areas, a goal which has been impossible to achieve with the current resolution offered by light isotopes alone. Additional sampling over a wider range of sites with different geological histories and bedrock types will likely help refine the predictive power of ^87^Sr/^86^Sr and could allow isotopic patterns for animals to be inferred from soil or plants. Nevertheless, our results demonstrate how isotopes influenced by vastly different global-scale processes can be used effectively to track long-distance animal movement.

## Supporting Information

Appendix S1 (0.03 MB XLS)

Appendix S2 (0.02 MB XLS)
